# Supplementary material for: A combined test for feature selection on sparse metaproteomics data—an alternative to missing value imputation
Source: PeerJ. 2022 Jun 24;10:e13525. doi: 10.7717/peerj.13525 (PMC9235818; doi:10.7717/peerj.13525)
Supplement: Supplemental Information 1 [file peerj-10-13525-s001.pdf]

A combined test for feature selection on sparse metaproteomics data - an alternative to missing value imputation

S. Plancade, M. Berland, M. Blein-Nicolas, O. Langella, A. Bassignani, C. Juste

1 Permutation procedure for the combined test

Preliminary: define the permutation design

According to the study design, the user can provide constraints on the permutations *via* control parameters defined by the function `how` to be passed to the R function `shuffle` (`permute` package). For the *ProteoCardis* datasets, no constraints were considered, but for *Pigs*, classes were permuted while keeping together the samples from the same animal.

Permutation test p-values

Let n be the number of biological samples in the dataset, and m the number of proteins. Let $(a_j)_{j=1,\ldots,m}$ be the number of non-missing intensities among the n samples for each protein $j = 1, \ldots, m$. After filtering of proteins with less than τ non-missing values, $a_j \in \{\tau, \ldots, n\}$ for all j. Then, for each $a \in \{\tau, \ldots, n\}$,

- Let $\mathcal{C}_a$ be the set of proteins with a non-missing values.
- For each protein $j \in \mathcal{C}_a$, classes are permuted repeatedly according to the chosen permutation design, for repetitions $r = 1, \ldots, \lfloor N^{perm}/\#\mathcal{C}_a \rfloor$ with $\lfloor \cdot \rfloor$ the integer part and $\#$ the cardinal, and the Fisher combined statistic $S^a_{j,r}$ is computed.
- The vector $(S^a_{j,r})_{j\in\mathcal{C}_a, r=1,\ldots,\lfloor N^{perm}/\#\mathcal{C}_a \rfloor}$ represents a sample of the distribution of the test statistic under the null hypothesis of no class effect, for proteins with a non-missing values.

Then, for each protein $j = 1, \ldots, m$, the p-value of the combined test is equal to:

$$p_j = \frac{1}{\#\mathcal{C}_{a_j} \times \lfloor N^{perm}/\#\mathcal{C}_{a_j} \rfloor} \sum_{j\in\mathcal{C}_{a_j}} \sum_{r=1}^{\lfloor N^{perm}/\#\mathcal{C}_{a_j} \rfloor} \mathbb{1}_{S_j > S^{a_j}_{j,r}} \quad \text{(s1)}$$

with S^j the Fisher combined statistics of protein j computed with the true classes.

Resampling based FDR

Resampling-based FDR is computed for 100 permutations. For $s = 1, \ldots, 100$,

- Classes are permuted according to the chosen permutation design.
- The Fisher combined statistic $(\widetilde{S}^s_j)_{j=1,\ldots,m}$ is computed, using the same permuted classes for all proteins.
- The vector $(p^{perm,s}_j)_{j=1,\ldots,m}$ of p-values under the complete null assumptions are computed by equation (s1) with S_j replaced by $\widetilde{S}^s_j$. Note that the distribution under the null assumption does not require to be computed again.

Following the procedure by Reiner et al. (2003), new estimates of the p-values are computed assuming that the marginal distributions under the complete null hypothesis are exchangeable:

$$p_j^{FDR} = \frac{1}{100m+1}\left(\sum_{\ell=1}^{m}\sum_{s=1}^{100} 1\!\!1_{p_\ell^{perm,s}\leq p_j} + 1\right)$$

Finally, FDR adjustment (Benjamini and Hochberg, 1995) is applied to $(p_j^{FDR})_{j=1,\ldots,m}$.

2 Simulation framework

General procedure

- Protein intensities from the data set *ProteoCardis-cyto* were filtered at threshold 10 (i.e. proteins with less than 10 non-missing values were removed), resulting in 11,433 proteins and 74% of missing values. Then the missing values were imputed by kNN, providing a realistic metaproteomic data set.
- Two classes of size 49 and 50 were randomly sampled among the 99 samples.
- 2000 proteins were randomly selected to be different between the two classes. Two types of difference were considered: (i) Differential intensity, (ii) Differential presence (see details below).
- Two missingness scenarios were considered: (ii) MAR: Missing values were drawn randomly such that the proportion of missing values on the total data set is equal to the proportion in the original data set *Proteocardis-cyto* after filtering at level 10; (ii) MNAR: a hard thresholding was applied, with threshold chosen to have the same proportion of missing values than on *ProteoCardis-cyto* after filtering at level 10.
- For the 2×2 scenarios, proteins with less than 20 non-missing values were removed, then the three FSMs SVD-lmm, single-lmm and the combined test were implemented, and the ROC curves were computed. Note that KNN-lmm was not considered since it includes the same imputation method used to generate the data set; Besides, this method has been shown to perform similarly to SVD-lmm.

Generate difference between groups

- **Differential intensity**. For each of the 2000 proteins, the quantity $FC_j/2$ was added to the intensities of samples from one class and substracted to the intensities of samples from the other class. The fold change FC_j was tuned so that the corresponding p-value of a t-test was approximately equal to $\alpha = 10^{-3}$, according to the standard deviation σ_j of the intensities of each protein. More precisely, for a fold-change FC_j, the t-test statistic is equal to

  $$S = \frac{FC_j}{\sqrt{\sigma_j^2/50 + \sigma_j^2/49}} \simeq \frac{5FC_j}{\sigma}$$

  Thus, setting the p-value to α is equivalent to:

  $$1 - F_{st}(S, \mathrm{df} = 97) = \alpha \quad \Rightarrow \quad FC_j \simeq \frac{\sigma}{5} G_{st}(1-\alpha, \mathrm{df} = 97)$$

  where F_{st} and G_{st} denote the cumulative distribution function and the quantile function of the student distribution.

- **Differential presence.** For each of the 2000 proteins, each intensity was set to NA with probability τ in one class and $1-\tau$ in the other class. The parameter τ was tuned such that the p-value of the Fisher exact test for the average table:

	Present	Absent
Class 1	$\lfloor 50\tau \rfloor$	$50 - \lfloor 50\tau \rfloor$
Class2	$49 - \lfloor 49\tau \rfloor$	$\lfloor 49\tau \rfloor$

was equal to $\alpha = 10^{-3}$, where $\lfloor \cdot \rfloor$ denotes the integer part.

3 Supplementary figures and tables

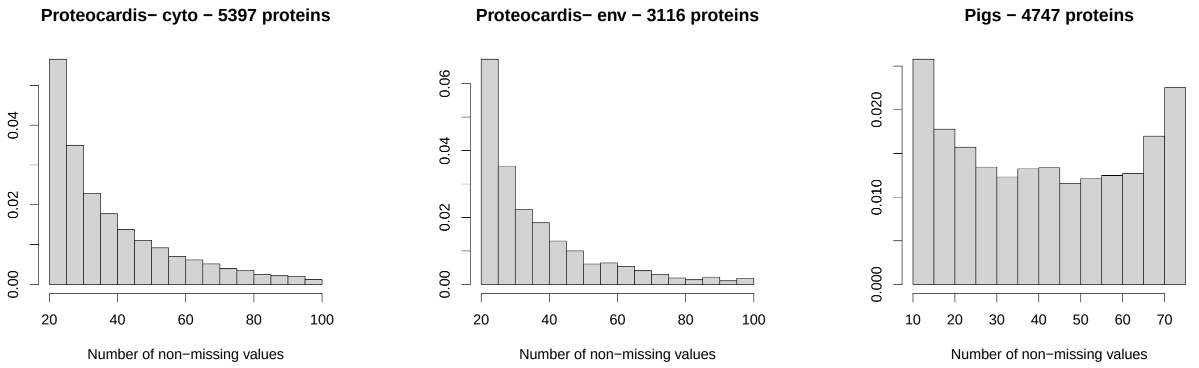

	Proteocardis-cyto					Proteocardis-env				
	Combined	KNN-lmm	Single-lmm	SVD-lmm	Hurdle	Combined	KNN-lmm	Single-lmm	SVD-lmm	Hurdle
q < 0.01	0	2	3	2	2	5	0	9	1	1
q < 0.05	6	2	27	3	15	13	5	17	2	29
q < 0.1	25	2	67	5	38	55	18	35	4	47
q < 0.2	92	5	223	23	125	113	82	80	6	118

	Pigs				
	Combined	KNN-lmm	Single-lmm	SVD-lmm	Hurdle
q < 0.01	1100	1205	1108	1310	914
q < 0.05	1831	1772	1754	1906	1669
q < 0.1	2289	2114	2176	2264	2125
q < 0.2	2867	2613	2666	2711	2605

Figure S1: **Statistical characteristics of the three data sets** *ProteoCardis-cyt*, *Proteocardis-env*, *Pigs*. Top: frequencies of the number of non-missing values for all proteins after filtering (threshold 20 for *ProteoCardis*, and 10 for *Pigs*). Bottom: number of selected variables with the resampling FDR procedure with 100 resampling repetitions, with various values of the FDR threshold values.

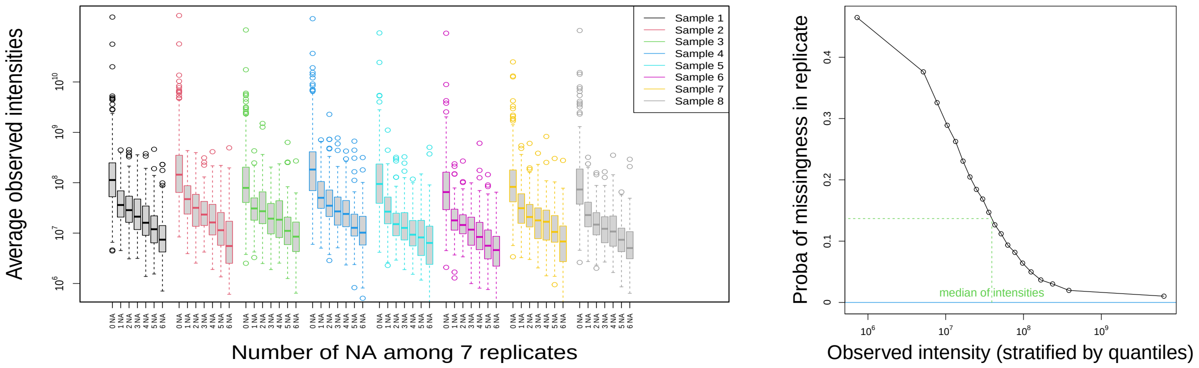

Figure S2: **Analysis of replicates - envelope fraction** Left: log10-transformed average intensities of non-missing observations, as a function of the number of missing values, for all proteins and for each biological sample. Right: Estimate of the probability that a protein is missing in a technical replicate as a function of the average of its non-missing values.

ProteoCardis-env

A - Correlation

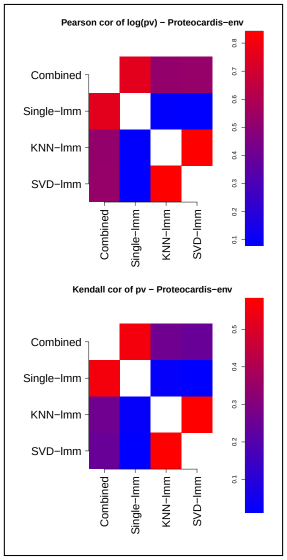

B - Proportion of common selected features

	Top 30			Top 100			Top 200		
	KNN-lmm	Single-lmm	SVD-lmm	KNN-lmm	Single-lmm	SVD-lmm	KNN-lmm	Single-lmm	SVD-lmm
Combined	0.30	0.33	0.37	0.43	0.50	0.41	0.39	0.56	0.40
KNN-lmm		0.07	0.53		0.11	0.67		0.15	0.67
Single-lmm			0.07			0.12			0.16

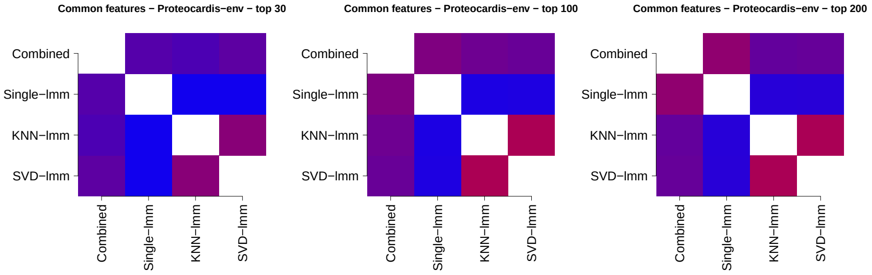

Figure S3: **Pairwise agreement between p-values of FSMs for** *Proteocardis-env***.** A: Pearson correlation between log of p-values and Kendall correlation between p-values . B: Proportion of common features among the top N ($N = 30, 100, 200$) for each pair of FSMs, as a table and a heatmap.

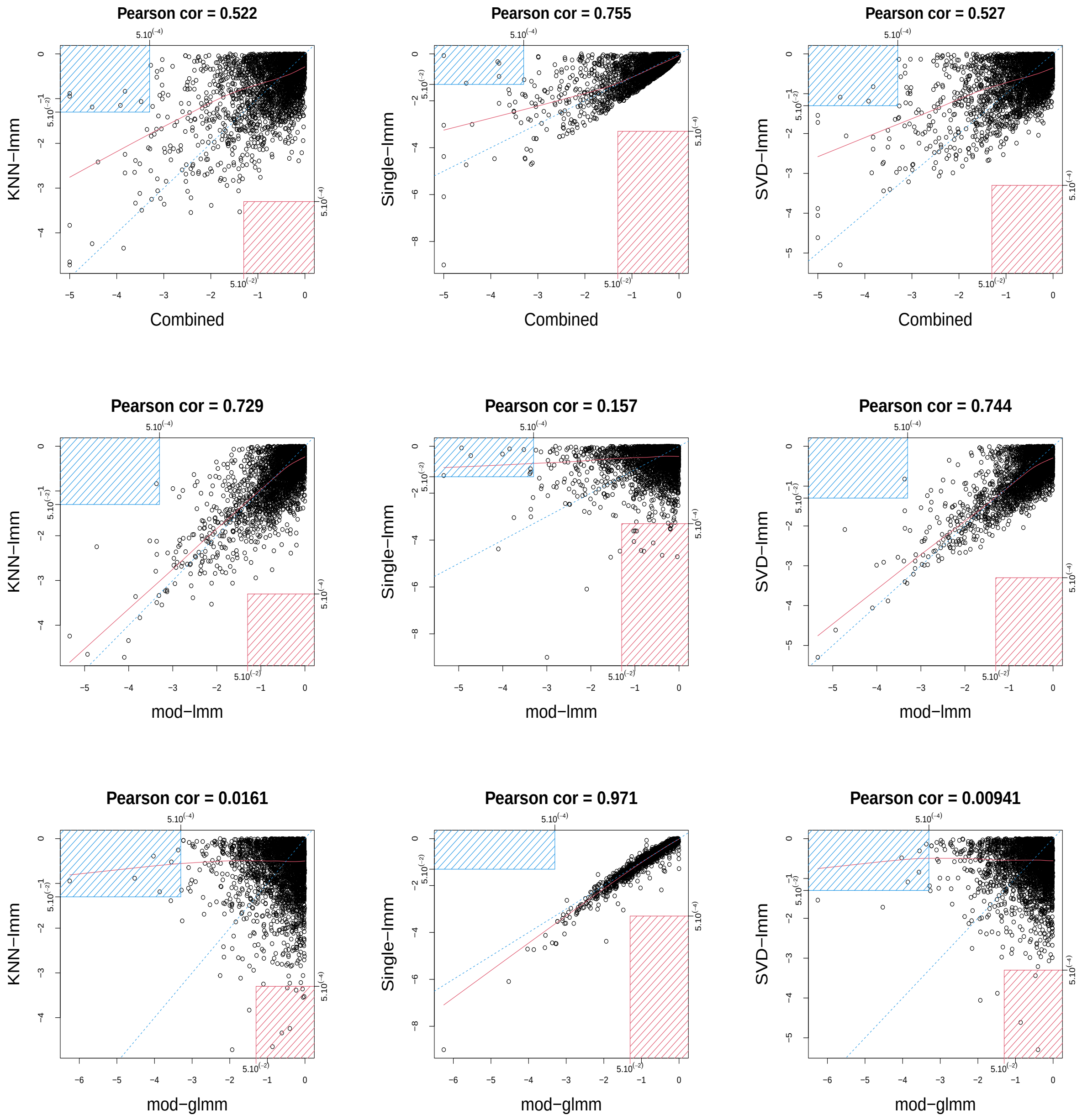

Figure S4: **Scatterplots between log10-transformed p-values of pairs of FSMs for** *Proteocardis-env.* Row 1: combined test and imputation-based FSMs. Row 2: Generalised mixed model (logistic) on missingness and imputation-based FSMs; proteins with less than 2 non-missing values are not displayed. Row 3: Linear mixed model on observed values and imputation-based FSMs. For each pair of testing procedure, the red rectangle corresponds to proteins with $p > 5.10^{-2}$ with the first procedure and with $p < 5.10^{-4}$ for the second procedure; conversely, the blue rectangle corresponds to proteins with $p < 5.10^{-4}$ with the first procedure and with $p > 5.10^{-2}$ for the second procedure.

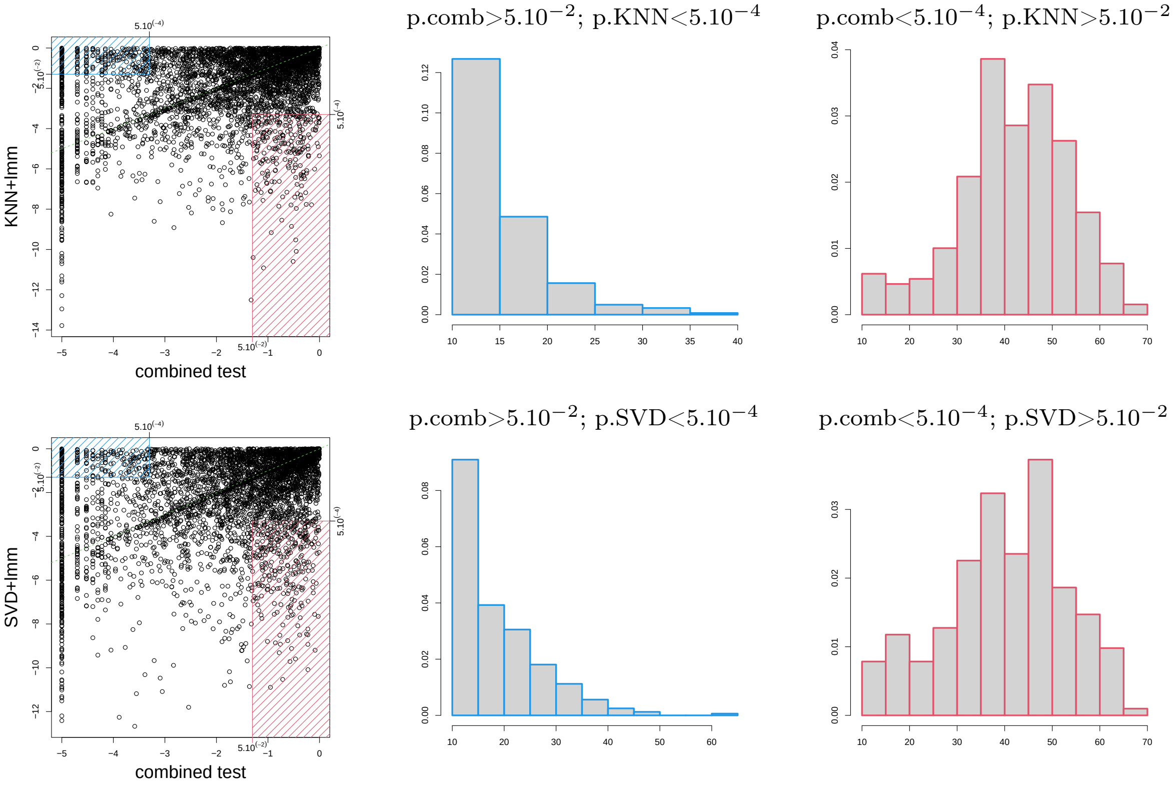

Figure S5: **Sparsity for proteins which are discordant** between the combined test and KNN-lmm (first row) or SVD-lmm (second row), on *Pigs*. Column 1: scatterplot of log10-transformed p-values of pairs of FSMs; the red rectangle corresponds to proteins with $p > 5.10^{-2}$ with the first procedure and with $p < 5.10^{-4}$ for the second procedure; conversely, the blue rectangle corresponds to proteins with $p < 5.10^{-4}$ with the first procedure and with $p > 5.10^{-2}$ for the second procedure. Column 2 (resp. 3): Histogram of the number of observed values by protein, for all proteins in the blue (resp. red) rectangle.

	**Proteocardis-cyto**			**Proteocardis-env**			**Pigs**		
	top30	top100	top200	top30	top100	top200	top200	top500	top1000
Combined	0.60	0.69	0.69	0.63	0.68	0.76	0.17	0.17	0.25
KNN-lmm	0.67	0.63	0.68	0.70	0.76	0.82	0.57	0.57	0.57
SVD-lmm	0.70	0.65	0.69	0.67	0.74	0.80	0.28	0.31	0.34
Single-lmm	0.60	0.68	0.72	0.60	0.69	0.78	0.60	0.57	0.54

Table S1: **Proportion of selected variables with less than half observed intensities**, among the top N variables (between 20 and 50 non-missing values for *Proteocardis* data sets, and between 10 and 36 for *Pigs*).

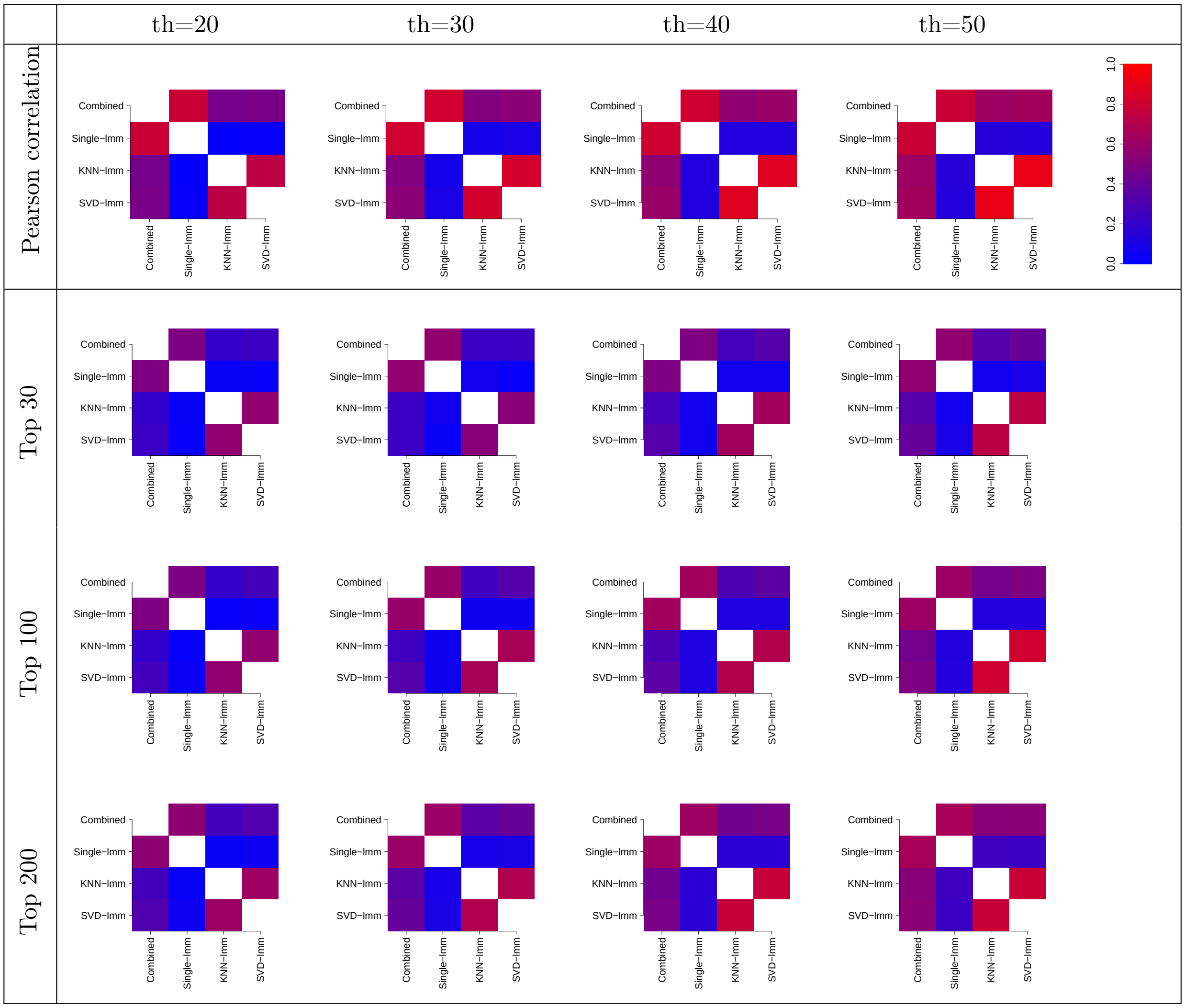

Figure S6: **Pairwise agreement between p-values from the four FSMs, for filtering threshold of 20, 30, 40 and 50 for** ***Proteocardis-cyto*****.** Each row correspond to a criterion; row 1: Pearson correlation between log-transformed p-values; rows 2 to 4: proportion of common variables among the top N variables with $N = 30, 100, 200$. Each column correspond to a threshold value.

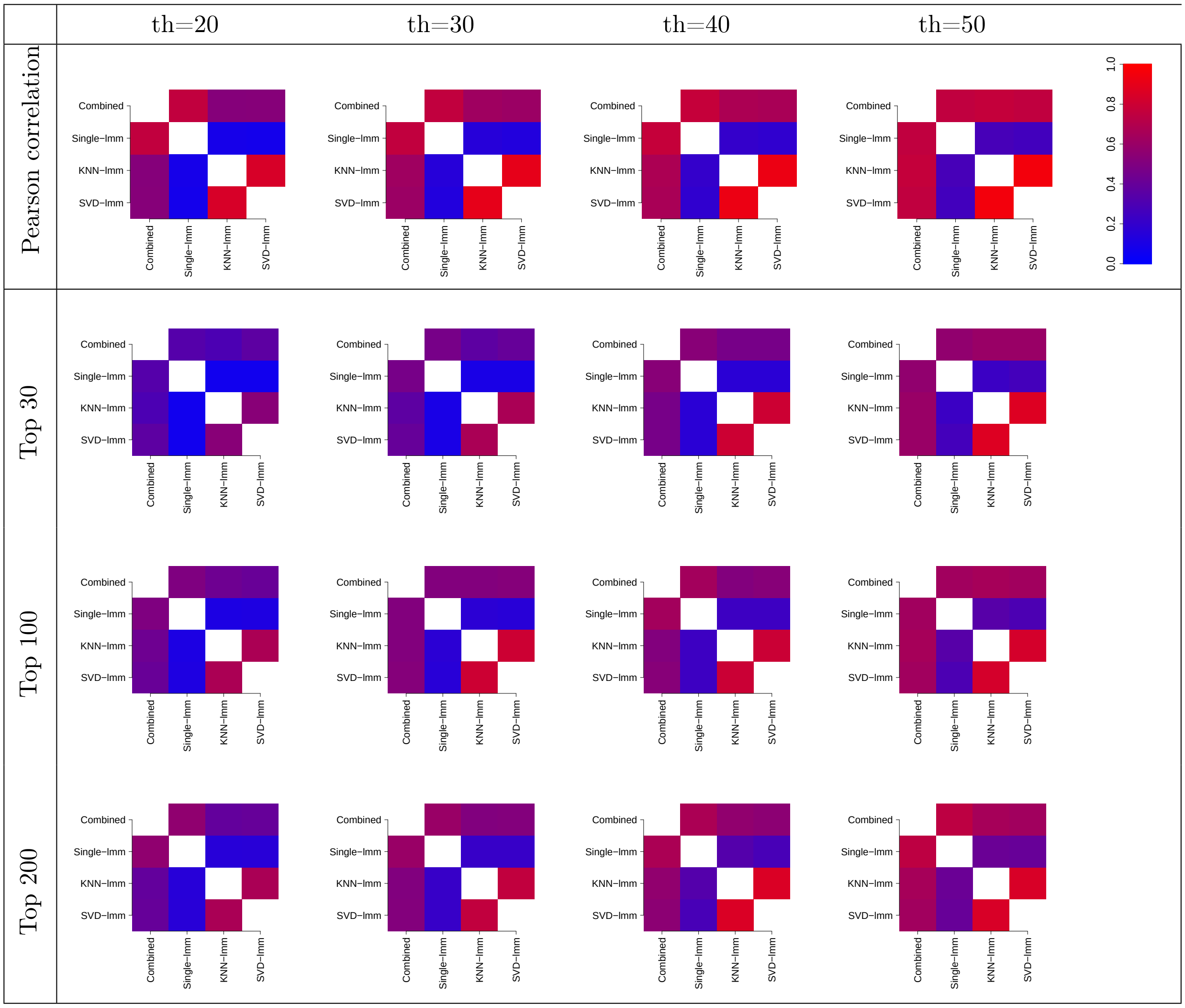

Figure S7: **Pairwise agreement between p-values from the four FSMs, for filtering threshold of 20, 30, 40 and 50 for** *Proteocardis-env.* Each row correspond to a criterion; row 1: Pearson correlation between log-transformed p-values; rows 2 to 4: proportion of common variables among the top N variables with $N = 30, 100, 200$. Each column correspond to a threshold value.

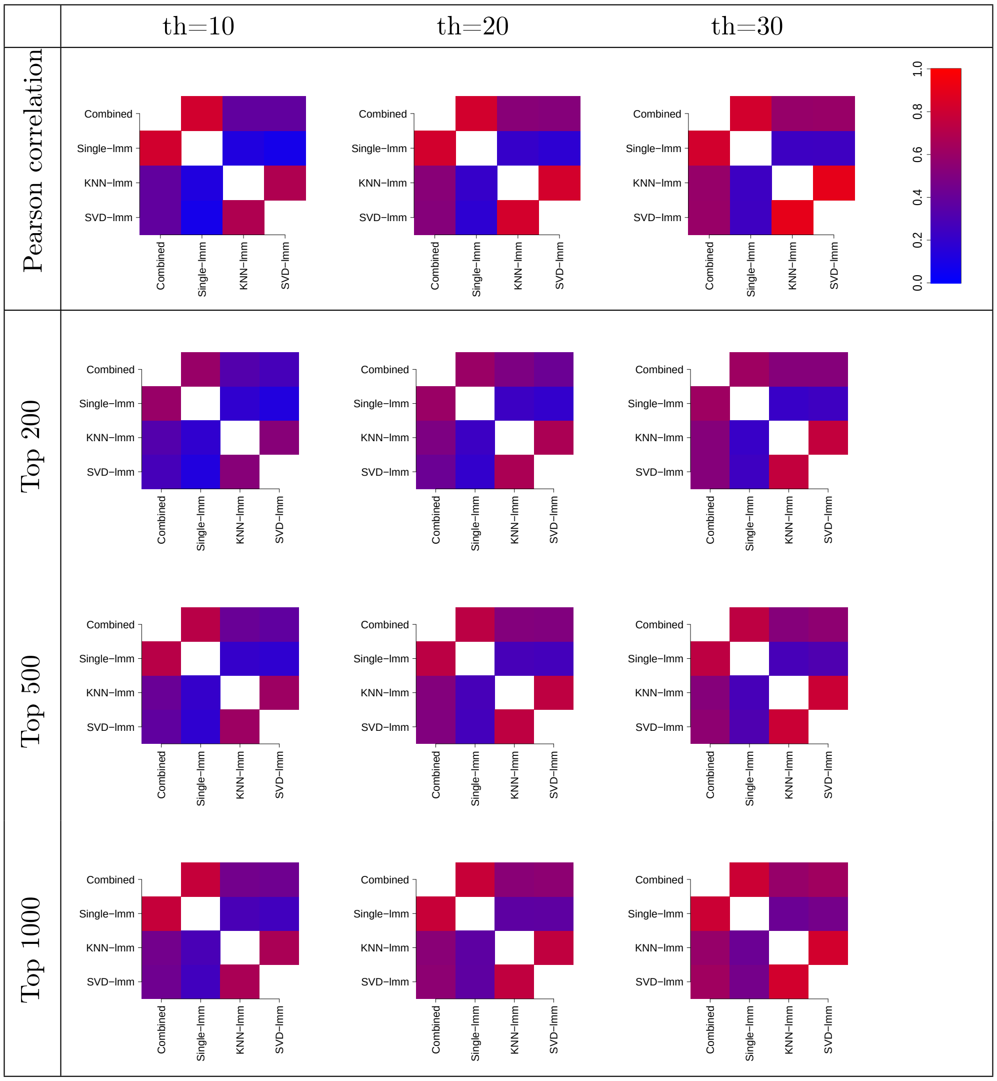

Figure S8: **Pairwise agreement between p-values from the four FSMs, for filtering threshold of 20 and 30 for *Pigs*.** Each row correspond to a criterion; row 1: Pearson correlation between log-transformed p-values; rows 2 to 4: proportion of common variables among the top N variables with $N = 200, 500, 1000$. Each column correspond to a threshold value.

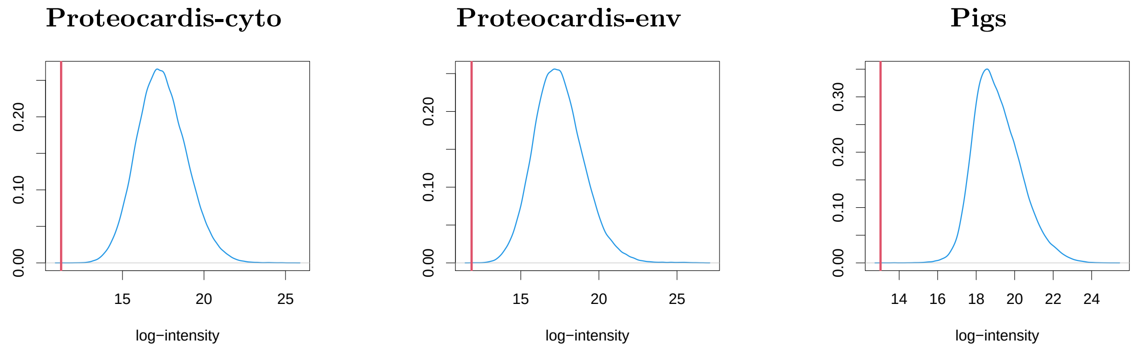

Figure S9: **Single value imputation.** Distribution of observed log-transformed intensities (blue) and imputed value (red) with single value imputation.

		FS combined test	FS KNN-lmm	FS SVD-lmm	FS single-lmm	FS hurdle test
Top 30	RF	**0.771**(0.021)	0.758(0.025)	0.719(0.017)	0.748(0.022)	0.767(0.017)
	SVM	0.748(0.025)	0.616(0)	0.668(0.0032)	0.734(0.0096)	**0.774**(0.013)
Top 100	RF	**0.769**(0.024)	0.761(0.013)	0.736(0.015)	0.741(0.017)	0.756(0.008)
	SVM	**0.772**(0.012)	0.73(0.022)	0.707(0)	0.741(0.013)	0.708(0.0032)
Top 200	RF	0.738(0.015)	0.737(0.017)	0.735(0.0093)	0.733(0.013)	**0.744**(0.015)
	SVM	**0.744**(0.012)	0.701(0.019)	0.681(0.019)	0.699(0.0064)	0.678(0.0032)

Table S2: **Prediction accuracy** for two classification procedures on *Proteocardis-env*. The selection of the top N variables ($N = 30, 100, 200$) was followed by SVM or RF. Accuracy was computed in a 10-fold cross validation loop, repeated 10 times. Each cell provides the average accuracy (standard deviation of accuracy) computed over the 10 repetitions of the cross-validation. Bold numbers correspond to the highest accuracy among the four FSMs

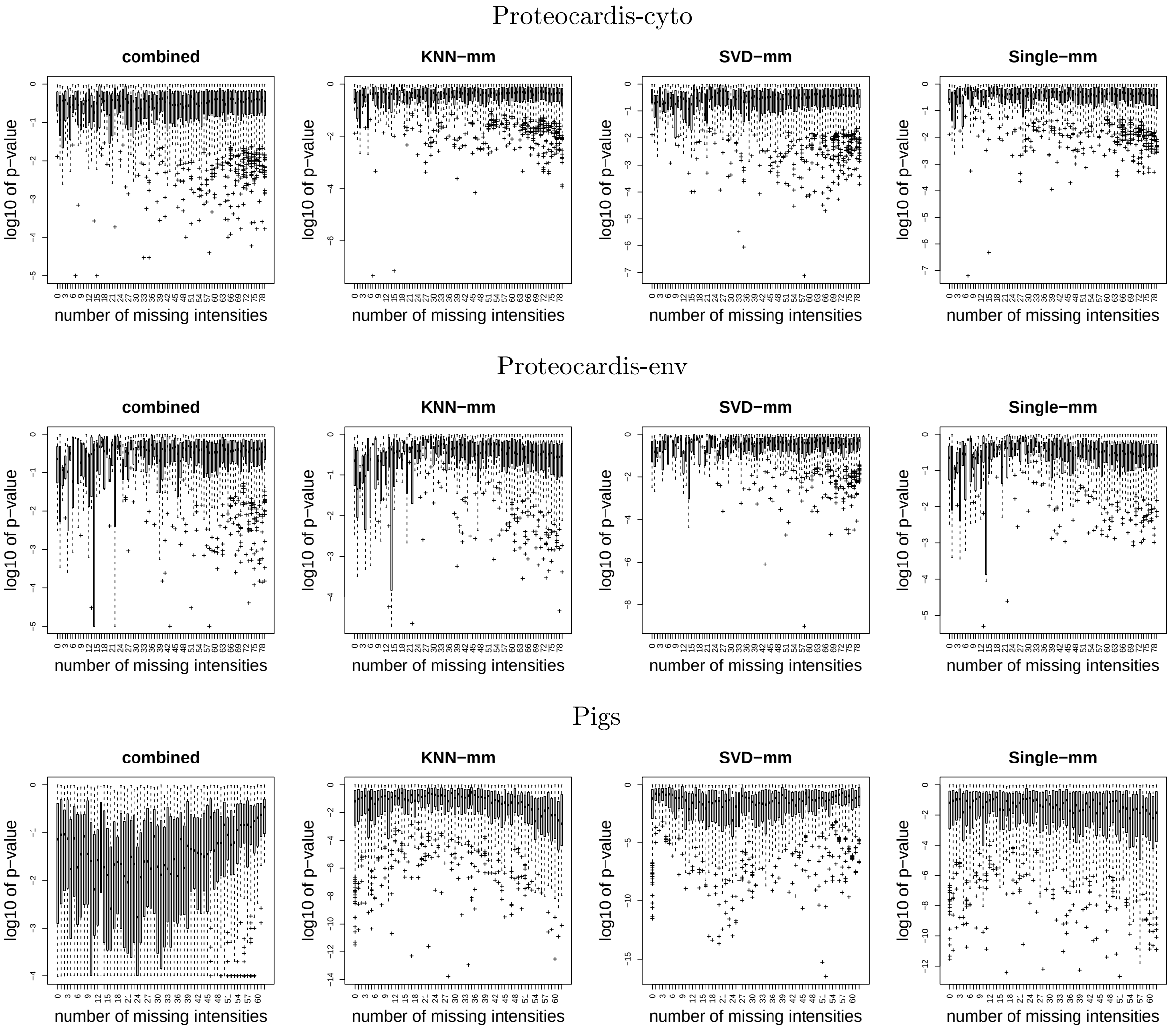

Figure S10: **Log10-transformed p-values as a function of sparsity**. The x-axis corresponds to the number of missing values among the 99 samples for *ProteoCardis* data sets, and among the 72 samples for *Pigs*.

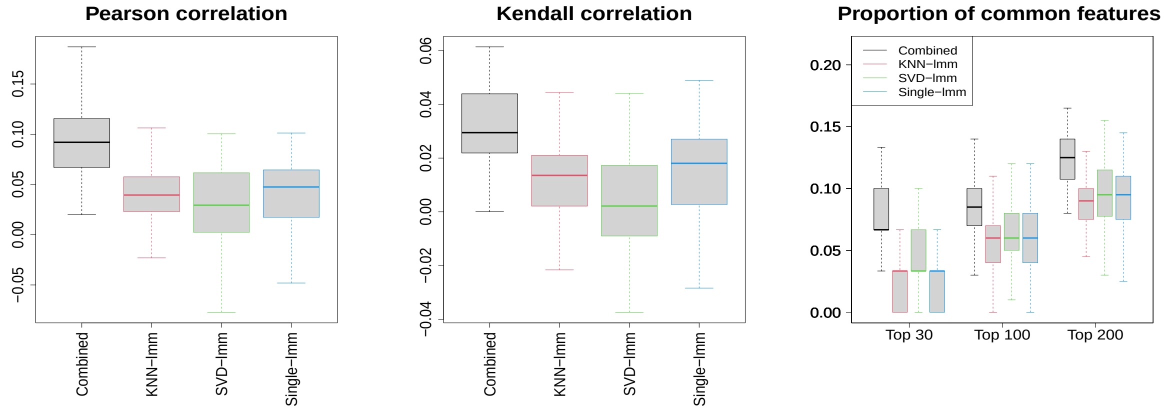

Figure S11: **Replicability of variable selection on independent subsets.** Pearson correlation between log-transformed p-values, Kendall correlation between p-values and proportion of common variables among the top N for 100 splitting of samples into two subsets. Dataset: *Proteocardis-env*.

Proteocardis-env

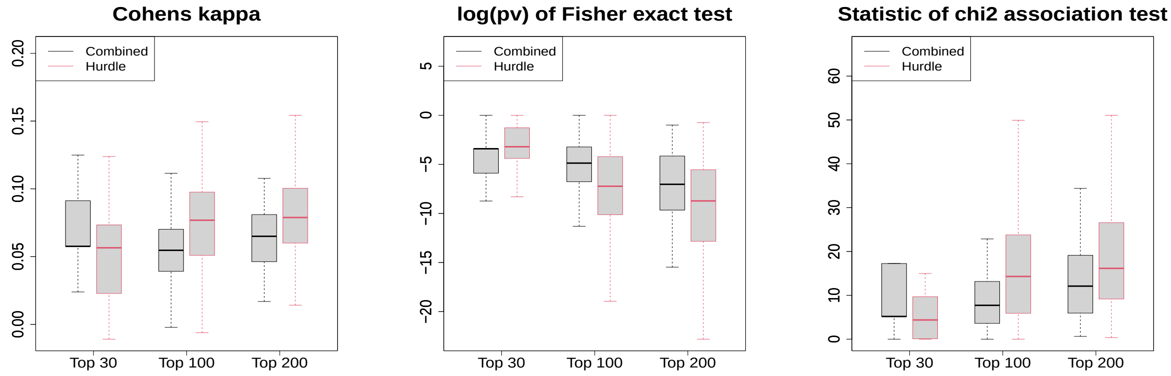

Figure S12: **Replicability of variable selection on independent subsets for the hurdle test and the combined test.** Boxplot of the Cohen's kappa (left), the log-transformed p-value of Fisher test (center) and the statistic of the χ^2 contingency table test (right), for selection of the top N features, performed on 100 splitting of the samples into two subsets. Black and red boxlots correspond to feature selection with the combined and the hurdle test respectively. Dataset: *ProteoCardis-env*

References

Benjamini, Y. and Hochberg, Y. (1995). Controlling the False Discovery Rate: A Practical and Powerful Approach to Multiple Testing. Journal of the Royal Statistical Society. Series B (Methodological), **57**(1), 289–300.

Reiner, A., Yekutieli, D., and Benjamini, Y. (2003). Identifying differentially expressed genes using false discovery rate controlling procedures. Bioinformatics, **19**(3), 368–375.
